# Screening for Breast Cancer: A Comparative Review of Guidelines

**DOI:** 10.3390/life14060777

**Published:** 2024-06-19

**Authors:** Laskarina Katsika, Eirini Boureka, Ioannis Kalogiannidis, Ioannis Tsakiridis, Ilias Tirodimos, Konstantinos Lallas, Zoi Tsimtsiou, Themistoklis Dagklis

**Affiliations:** 1 Laboratory of Hygiene, Social & Preventive Medicine and Medical Statistics, School of Medicine, Faculty of Health Sciences, Aristotle University of Thessaloniki, 541 24 Thessaloniki, Greece; laskarina.katsika@gmail.com (L.K.); ityrodim@auth.gr (I.T.); ztsimtsiou@auth.gr (Z.T.); 2 Third Department of Obstetrics and Gynecology, School of Medicine, Faculty of Health Sciences, Aristotle University of Thessaloniki, 541 24 Thessaloniki, Greece; rboureka@gmail.com (E.B.); ikalogia@auth.gr (I.K.); dagklis@auth.g (T.D.); 3 Department of Medical Oncology, School of Medicine, Aristotle University of Thessaloniki, 541 24 Thessaloniki, Greece; koplallas@gmail.com

**Keywords:** breast cancer, mammography, screening, guidelines, comparison

## Abstract

Breast cancer is the most common malignancy diagnosed in the female population worldwide and the leading cause of death among perimenopausal women. Screening is essential, since earlier detection in combination with improvements in breast cancer treatment can reduce the associated mortality. The aim of this study was to review and compare the recommendations from published guidelines on breast cancer screening. A total of 14 guidelines on breast cancer screening issued between 2014 and 2022 were identified. A descriptive review of relevant guidelines by the World Health Organization (WHO), the U.S. Preventive Services Task Force (USPSTF), the American Cancer Society (ACS), the National Comprehensive Cancer Network (NCCN), the American College of Obstetricians and Gynecologists (ACOG), the American Society of Breast Surgeons (ASBrS), the American College of Radiology (ACR), the Task Force on Preventive Health Care (CTFPHC), the European Commission Initiative on Breast Cancer (ECIBC), the European Society for Medical Oncology (ESMO), the Royal Australian College of General Practitioners (RACGP) and the Japanese Journal of Clinical Oncology (JJCO) for women both at average and high-risk was carried out. There is a consensus among all the reviewed guidelines that mammography is the gold standard screening modality for average-risk women. For this risk group, most of the guidelines suggest annual or biennial mammographic screening at 40–74 years, while screening should particularly focus at 50–69 years. Most of the guidelines suggest that the age limit to stop screening should be determined based on the women’s health status and life expectancy. For women at high-risk, most guidelines recommend the use of annual mammography or magnetic resonance imaging, while the starting age should be earlier than the average-risk group, depending on the risk factor. There is discrepancy among the recommendations regarding the age at onset of screening in the various high-risk categories. The development of consistent international practice protocols for the most appropriate breast cancer screening programs seems of major importance to reduce mortality rates and safely guide everyday clinical practice.

## 1. Introduction

Breast cancer constitutes a major public health concern; it is the most common malignancy diagnosed in the female population worldwide and is characterized by high morbidity and mortality [1]. The incidence of breast cancer is approximately 145 per 100,000 with a mortality of 33 per 100,000 women per year [2]. One in eight women is expected to develop breast cancer in their lifetime and it is the leading cause of death among women aged 45–55 years [3].

There are many factors involved in the process of carcinogenesis, with age and female gender being the major risk factors for breast cancer [4]. Other known risk factors related to breast cancer include history of certain types of malignancies (breast, ovarian, pancreatic), high breast density, history of chest irradiation in a young age, genetic mutations (BRCA 1, 2 genes), history of breast pathology (atypical hyperplasia, lobular carcinoma in situ—LCIS), alcohol consumption and smoking, obstetric and gynecological factors (early menarche, late menopause, nulliparity, menopausal hormone therapy) and increased body mass index [4].

Screening is of great value since early detection combined with improvements in breast cancer treatment may reduce the associated mortality [5]. Guidelines are based on the best available scientific evidence and their provision, both at national and international level, contributes to improving quality of care. However, they are often complex and present with several discrepancies. Therefore, the development and implementation of uniform international evidence-based algorithms for the most appropriate breast cancer screening methods is of outmost importance to reduce its incidence and the overall mortality rate. Hence, the aim of this descriptive review was to summarize and compare recommendations from influential guidelines on breast cancer screening.

## 2. Evidence Acquisition

The most recently published guidelines on the screening of breast cancer were retrieved and a descriptive review was conducted. In particular, 12 relevant guidelines were identified from the World Health Organization (WHO) [5], the U.S. Preventive Services Task Force (USPSTF) [6], the American Cancer Society (ACS) [7], the National Comprehensive Cancer Network (NCCN) [8], the American College of Obstetricians and Gynecologists (ACOG) [4], the American Society of Breast Surgeons (ASBrS) [9], the American College of Radiology (ACR) [10], the Task Force on Preventive Health Care (CTFPHC) [11], the European Commission Initiative on Breast Cancer (ECIBC) [12], the European Society for Medical Oncology (ESMO) [13], the Royal Australian College of General Practitioners (RACGP) [14] and the Japanese Journal of Clinical Oncology (JJCO) [15].

This comparative review of the aforementioned guidelines was conducted based on the risk of developing breast cancer. The recommendations were classified in involving average or high-risk women and their overview is presented in the corresponding two tables (Table 1 for average and Table 2 for high-risk women).

## 3. Screening Recommendations for Women at Average Risk

### 3.1. Recommendations for Women at 25–39 Years

In the 25–39 age range, three guidelines (ACOG, ASBrS, NCCN) list recommendations for initiation of screening, with ASBrS differentiating recommendations further for the 25–30 and 30–40 age groups. ACOG and NCCN suggest initiation of clinical breast examinations (CBE) from the age of 25 and re-evaluation every 1 to 3 years. This recommendation is based on low-quality studies that showed that approximately 2–6% of breast cancers were identified by CBE alone and were missed from the mammography screening [16,17,18]. However, these studies were conducted at a time when the technology and sensitivity of mammography was lower than in recent years and therefore present a high risk of bias. Nevertheless, ACOG and NCCN suggest CBE at this specific age group, provided that women are informed of the risk of false-positive results and the possibility of unnecessary further examinations. ASBrS, on the other hand, suggests early screening with risk assessment for every age group and subsequent follow-up after classification. More specifically, for women aged 25–39 years with an overall average risk for breast cancer development, no further screening is required, apart from a regular repeat of the risk assessment evaluation. Regarding the frequency of screening in this age group, ACOG and NCCN recommend an interval from 1 to 3 years, while ASBrS does not provide further information.

### 3.2. Recommendations for Women at 40–49 Years

For women between the ages of 40 to 49 years the reviewed guidelines present with major controversies. More specifically, ACOG, ACR ASBrS, NCCN, USPSTF and JJCO agree on the initiation of screening with mammography after the age of 40 with a screening interval every 1 to 2 years (ACOG), annually (ACR, ASBrS, NCCN) or biennial (USPSTF). This recommendation is based on studies demonstrating a benefit from early initiation of screening as, despite the fewer cases detected, it prevents a significant number of cancer-related deaths [19]. However, these medical societies point out the necessity of an informed decision before initiation of screening, as there is an increased number of false-positive results and possible unnecessary procedures and outcomes (biopsies, further screening, increased anxiety) [19,20]. As for the optimal screening interval, the above-mentioned recommendations are based on a large study that showed an overall benefit from annual screening on detection rates and life-years gained but an additional significant increase in unnecessary biopsies, call-backs and psychosocial burden [21]. Therefore, some societies prefer to adopt an annual and some a biennial approach. Based on the same studies, ESMO and RACGP state against universal screening for women aged 40 to 49 years, but mention that it can be performed in an individual basis, after careful assessment of the patient’s history and risk factors. Moreover, ACS and ECIBC recommend initiation of screening at the age of 45 due to several data indicating an ascendancy on the benefit gained from early screening compared to any possible harms caused [22,23]. Interestingly, controversy exists regarding the time intervals, with ACS suggesting annual, whereas ECIBC suggests biennial or triennial screening programs. On the other hand, WHO and CTFPHE advocate against routine mammography screening in the age group of 40 to 49 years; WHO recommends it only in the context of rigorous research, in well-resourced settings. This recommendation is based on global cancer data which demonstrate that in this age group, the incidence of breast cancer is significantly lower than after the age of 50 [24,25]. Therefore, considering the non-cost-effectiveness character of such policy and the effect on low-income countries, they state against it overall.

### 3.3. Recommendations for Women at 50–69 Years

There is agreement on the appropriate screening program for the age range of 50 to 69 years, with all the reviewed guidelines recommending the use of mammography. This recommendation is based on several studies that showed that the overall benefit from screening in this age group overcomes any possible harm [19,20]. More specifically, data from several trials showed that when implementing a well-organized screening program, there was an overall reduction in deaths due to breast cancer, as well as a reduction in diagnosis of advanced stages [26,27,28]. Despite the agreement from the reviewed medical societies on the appropriate screening method, controversy exists regarding the screening intervals. Some of the guidelines recommend annual (ACR, ASBrS, NCCN), others recommend biennial screening (WHO, USPSTF, ECIBC, RACGP) and others recommend every one to two years (ACOG, ACS, ESMO). Interestingly, CTFPHC is the only medical society suggesting mammography screening every two to three years. These recommendations are all based on the same above-mentioned studies, as mentioned for the age group of 40 to 49 years.

### 3.4. Recommendations for Women at 70–74 Years

All the reviewed guidelines, except from WHO, agree regarding the optimal screening with mammography for the age group of 70 to 74 years. This recommendation is based on several studies that showed a significant decrease in breast-related adverse events (mortality, low quality-adjusted-life-years) when implementing a screening strategy in this particular age group, compared to no screening [21,27,28,29]. However, they mention that these benefits apply only for patients with an average good health status, low number of comorbidities and an overall adequate life expectancy. WHO, on the other hand, suggests implementing mammography screening only in well-resourced research settings, whereas it states against screening in low-income countries. Following the recommendations for the previous age groups, the reviewed guidelines present with several controversies regarding the check-up intervals. It is worth mentioning that most of the medical societies follow the guidelines they issued in earlier age ranges, except from WHO that is against screening and ECIBC that recommends mammography screening every 3 years, while discouraging annual mammography screening. The ECIBC recommendation is based on a study of 100,000 women which showed that, for this age group, the cumulative effect of radiation from mammography screening results in 125 new breast cancer cases and 16 cancer-related deaths [30]. However, apart from WHO and ECIBC, the rest of the medical societies are based on several studies that show a higher incidence of cancer cases diagnosed in advanced stages following biennial screening, versus annual (OR: 1.6; 95% CI: 1.0–2.5), but taking into account the adverse outcomes of annual screening (radiation, false-positive results, fewer call-backs) and the economic status and financial policy of each country, both annual and biennial approach are considered acceptable [27,31,32,33].

## 4. When to Stop Screening

There is an overall agreement that the appropriate age to stop screening has not been established; all the medical societies that make recommendations on this issue mention that there are insufficient data to support an evidence-based recommendation. This is mainly because all the reviewed trials do not include women above the age of 74; therefore, the only data regarding this age group originate, mostly, from small observational studies. Nevertheless, seven from the reviewed guidelines (ACOG, ACR, ACS, ASBrS, NCCN, USPSTF, RACGP) are in line regarding the most appropriate timing to stop screening. They state that after the age of 75, the decision to stop mammography screening should be based on a shared decision-making process based on the woman’s general health and life expectancy. More specifically, in women with a good overall health status and a life expectancy of more than 10 years, ACOG, ACR, ACS, ASBrS, NCCN, USPSTF and RACGP support the continuation of mammography screening, when in line with the patient’s wishes, with either annual (ACR, ASBrS) or biennial (USPSTF) screening. This recommendation is based on data that showed that approximately one out of four breast cancer deaths will be due to a diagnosis after the age of 74 [34], and the overall sensitivity of mammography screening is most likely to improve with age, as fewer false-positive results are expected [35].

Moreover, the overall patient’s condition should be considered, especially in cases where any possible cancer diagnosis and treatment would aggravate their general health status, even in ages younger than 75 years, who are complicated with severe comorbidities. This recommendation is based on a study of almost 90,000 cases with severe comorbidities that showed that, despite low life expectancy, a policy of continuing screening was followed [36]. The results, however, failed to demonstrate any significant benefit from screening policy, but caused, nevertheless, an increasing amount of anxiety and psychological burden, in anticipation of the results [36].

Therefore, the optimal timing for screening cessation should consider the overall health status of the patient, the life expectancy based on comorbid conditions, the age group and the informed decision of the patient.

## 5. Other Screening Methods

All the reviewed guidelines make recommendations regarding alternative screening methods, such as CBE, ultrasonography and magnetic resonance imaging (MRI).

Regarding CBE, there is a general agreement against its use as a screening method in average-risk women. More specifically, ACOG, ACS, CTFPHC, RACGP and JJCO state that it should be offered only following carefully informing of the patient about its restrictions and controversies regarding its predictive value. This recommendation is based on low-quality data that demonstrated a small contribution (3–6%) on breast cancer diagnosis from the combination of CBE and mammography but no actual benefit to the patient’s outcome [16,37]. WHO and NCCN, on the other hand, suggest screening with CBE in women between the ages of 40 and 69, in low-income countries, where mammography screening is not feasible (WHO), or in the general population, annually, after the age of 40 and every 1 to 3 years in the age group of 25–39 (NCCN). This is based on a large trial that showed an overall sensitivity and specificity of 51.7% and 94.3%, respectively, with a higher detection rate in advance-stages of cancer (stage IIB or higher) [38].

Moreover, with regards to ultrasonography, USPSTF, ECIBC, ESMO and JJCO are against its use as a screening method for breast cancer, whereas CTFPHC accepts its use only as a supplementary tool to mammography. More specifically, ECIBC points out the uncertainty of the predictive value of a supplementary or single sonographic examination and the additional down-effects from overdiagnosis and regular call-backs, as well as the overall economic burden to the healthcare system. Additionally, JJCO is questioning the test’s accuracy and underlines the lack of adequate data to support its use in every-day clinical practice. The previous recommendations are based on several studies that showed a small increase in breast cancer detection when combining mammography with ultrasound examination, but with a more significant increase in false-positive results, unnecessary biopsies and more call backs [39,40,41,42]. Moreover, a study of almost 3000 women showed that when combining mammography with ultrasound the overall recall rate increased to 14% [43]. The only medical society in favor of ultrasound is NCCN that recommends clinical encounter every 1 to 3 years between the ages of 25 to 39 years and annually after the age of 40. By clinical encounter, NCCN means screening with a series of examinations, including CBE and evaluation of risks factors, mammography, breast MRI and sonography.

Screening with MRI is a topic discussed by fewer medical societies, but with statements similar to the ones for sonography; only ACR, USPSTF and ECIBC make relevant recommendations, whereas NCCN refers only to women at high risk of breast cancer. More specifically, ACR, USPSTF and ECIBC point out the lack of sufficient data to support or discourage the implementation of MRI screening, while also underlining the possible harms from overdiagnosis. Moreover, ECIBC is the only medical society raising the issue of cost effectiveness in stating against routine MRI as an addition to mammography. These recommendations are based on a trial of almost 3000 women that underwent screening with MRI, ultrasound and mammography examination; in the group of patients screened with a combination of all three examinations the sensitivity was 100% and the specificity was 65%. Moreover, the number needed to treat for MRI examination after negative mammography and ultrasound result was 68 [43]. These results highlight an increased number of cancer cases diagnosed after the use of MRI, but with a higher rate of false-positive results [43]. Apart from the aforementioned trial, several observational studies have demonstrated similar results, reaching the same conclusion regarding MRI use in real-life practice [44,45]

## 6. Screening Recommendations for Women at High-Risk

A total of four relevant guidelines published between 2018–2022 were studied. Three are from American Organizations (ACR, ASBrS, NCCN) and one is from the European Society for Medical Oncology (ESMO).

### 6.1. Women with a Lifetime Risk of Breast Cancer > 20% Based on Models Largely Dependent on Family History

Only two of the aforementioned medical societies make references regarding this specific category of patients (ASBrS, NCCN); according to ASBrS, women in this category should be encouraged to initiate annual screening with mammography and simultaneous complementary imaging (preferred MRI). This recommendation is based on a study that showed that MRI sensitivity in this group of patients was 90.0% compared to 37.5% for mammography and ultrasound examination [46]. Likewise, NCCN recommends annual digital mammography, preferably with tomosynthesis examination, beginning 10 years before the youngest affected family member but not prior to the age of 30. At the same time, it recommends annual screening with MRI, starting 10 years before the youngest affected family member but not before 25 years. Clinical evaluation is encouraged every 6 to 12 months, beginning at the age when the woman is identified as being at increased risk, but no earlier than the age of 21. Of note, NCCN mentions the inability to perform MRI screening in patients from low-income countries or rural areas.

### 6.2. Women Who Have a Lifetime Risk > 20% Based on History of Lobular Carcinoma In Situ or Atypical Ductal/Lobular Hyperplasia

Moreover, for women with a history of atypical ductal hyperplasia (ADH), atypical lobular hyperplasia (ALH) or lobular carcinoma in situ (LCIS) and a predicted risk of developing breast cancer of ≥20%, NCCN makes the same recommendations as above-mentioned. In addition, NCCN mentions that the sensitivity of MRI in detecting cancerous lesions in women with this history is questionable. This suggestion is based on a prospective study where the combination of mammography and MRI did not significantly improve the detection rate but increased the biopsy rate from 12.6% in screening with mammography alone, to 30.5% (HR: 2.67; *p* < 0.001) [47]. On the other hand, as there is a lack of sufficient data, NCCN suggests screening with a combination of both techniques, pending more evidence-based guidelines. Moreover, ACR recommends the same strategy, but without specifying the appropriate age criteria.

### 6.3. Women Who Have Received Prior Thoracic Irradiation at the Ages of 10 to 30 Years

Three of the reviewed medical societies make recommendations relating to women with prior thoracic irradiation (ACR, ASBrS, NCCN). They agree on the initiation of screening in these women no sooner than 25 years of age or 8 years post radiotherapy (RT). More specifically, NCCN recommends annual clinical evaluation in women aged less than 25 years after discontinuation of RT for 8 years. Additionally, NCCN lists detailed screening methods for ages 25 and older, recommending clinical evaluation every 6 to 12 months, starting 8 years after RT; annual mammography (digital breast tomosynthesis—DBT preferred) 8 years after radiation therapy but not before 30 years; and annual MRI 8 years after radiotherapy but not before 25 years. The same approach seems to be followed by ASBrS, which recommends the age of 25 as the milestone for commencement of MRI screening and 30 for mammography (both performed annually). On the other hand, ACR does not mention screening with MRI but recommends digital mammography or DBT every year starting at the age of 25, or 8 years after RT (whichever is later). These recommendations are based on data that take into account the increasing number of breast cancer cases between the ages of 25 to 30 years while comparing the diagnostic accuracy of mammogram and MRI and weighing the effect of cumulative radiation in these patients [48]. Results from a prospective study showed that MRI was less sensitive in diagnosing breast cancer, whereas the combination of modalities provided the best possible combination, reaching, therefore, to the conclusion that the overall risk of mammography radiation was negligible compared to the risk of undiagnosed cancer [49].

### 6.4. Women with a Known Genetic Predisposition

For women who have a high risk of developing breast cancer, due to pathogenic mutations, ACR, ASBrS and ESMO are in agreement. More specifically, ACR and ASBrS suggest annual MRI screening starting at the age of 25 and annual mammogram (preferably DBT) starting at the age of 30 as the appropriate method of screening. At the same time, ESMO is in line with the two above-mentioned guidelines, but it does not specify the optimal timing for initiation of screening. These recommendations are based on a systematic review that showed that the negative likelihood ratio was lower when combining mammography and MRI in patients with BRCA 1 and 2 mutations (0.14%; 95% CI: 0.05–0.42%), compared to mammography alone (0.70%; 95% CI: 0.59–0.82) defining as positive a BI-RADS score of 4 or more [50].

## 7. Conclusions

To summarize, the latest and most influential guidelines on breast cancer screening were comparatively evaluated. Guidelines for both average-risk and high-risk women were examined separately. The majority of guidelines contained similar recommendations regarding the age range, methods and intervals of screening. Specifically, most guidelines recommend annual or biannual screening with mammography for average-risk women aged 40–74 years, while for high-risk women, annual mammography or breast MRI is recommended starting at an early age based on the risk factor.

More specifically, in the 40–49 age range of average-risk populations, most organizations (except from CTFPHC) agree with mammography screening, albeit as a qualified recommendation, with most agreeing that it should be performed every 1–2 years. Before the age of 40, only three medical societies (ACOG, ASBrS, NCCN) have issued relevant recommendations, according to which self-awareness and CBE are encouraged, as well as continuous assessment of the risk of developing the disease. The age group of 50–69 years is considered optimal for screening, due to the increase in the incidence of breast cancer cases. All the reviewed guidelines agree that the mammography is the primary screening method for average-risk women. For women over the age of 70, continuation of screening is recommended with the use of mammography; in this particular age range, the possibility of screening cessation is considered due to factors such as the general state of the woman’s health and her life expectancy that should be taken into account very seriously in order to proceed, with an individualized decision-making process. For women at high risk of developing breast cancer, the guidelines are individualized based on the underlining risk factors. The main risk factors presented and associated with a higher risk of developing the disease (≥20%) are a strong family history; a history of chest radiotherapy at the age of 10–30 years; a history of ADH, ALH or LCIS; and genetic predisposition due to pathogenic mutations.

In conclusion, women should be extensively informed about the potential benefits, limitations and risks associated with breast cancer screening and a strong emphasis should be placed on shared decision making. International multicenter collaboration is needed to have guidelines based on strong scientific evidence, considering the different resource settings among low-, middle- or high-income countries. Especially in breast cancer cases, it is expected that the development of an international consensus on screening would contribute to an early diagnosis and eventually to a further reduction in women’s morbidity and mortality.

## Figures and Tables

**Table 1:** Breast cancer screening recommendations in average-risk women.

	WHO	ACOG	ACR	ACS	ASBrS	NCCN	USPSTF	CTFPHC	ECIBC	ESMO	RACGP	JJCO
**Country**	Global	United States of America	United States of America	United States of America	United States of America	United States of America	United States of America	Canada	Europe	Europe	Australia	Japan
**Issued**	2014	2017	2017	2015	2019	2022	2016	2017	2020	2019	2021	2016
**Title**	WHO position paper on mammography screening	Breast Cancer Risk Assessment and Screening in Average-Risk Women	Breast Cancer Screening for Average-Risk Women: Recommendations from the ACR Commission in Breast Imaging	Breast Cancer Screening for Women at Average Risk 2015 Guideline Update from the American Cancer Society	Position Statement on Screening Mammography	Breast Cancer Screening and Diagnosis	Screening for Breast Cancer: U.S. Preventive Services Task Force Recommendation Statement	Breast Cancer Screening: Protocol for an Evidence Report to Inform an Update of the Canadian Task Force on Preventive Health Care 2011 Guidelines	Breast Cancer Screening and Diagnosis: A Synopsis of the European Breast Guidelines	Early breast cancer: ESMO Clinical Practice Guidelines for diagnosis, treatment, and follow-up	Guidelines for preventive activities in general practice	The Japanese Guidelines for Breast Cancer Screening
**Pages**	82	16	7	16	10	89	19	66	12	27	382	11
**References**	37	55	151	122	24	222	62	103	107	216	144	85
	**25–39 years**											
**Screening methods**	NR	Clinical breast examination	NR	NR	25–30 years Risk assessment ≥30 years Risk assessment using the Tyrer–Cuzick	Clinical breast examination	NR	NR	NR	NR	NR	NR
**Screening intervals**	NR	Every 1–3 years	NR	NR	NR	Every 1–3 years	NR	NR	NR	NR	NR	NR
	**40–49 years**											
**Screening methods**	Mammography ▪ Well-resource settings: only for rigorous research ▪ Limited resource settings: not recommended	Clinical breast examination / Mammography (should initiate screening mammography no earlier than age 40 years but no later than age of 50 years)	Mammography (Should initiate screening mammography at age 40)	40–44 years Mammography 45–54 years Mammography (Screening mammography starting at 45 years)	Mammography (Screening mammography beginning at 40 years)	Clinical encounter / Mammography (DBT is recommended, if available)	Mammography Not as a routine	Screening is not recommended	40–44 years Screening is not recommended 45–49 years Mammography recommended	Mammography may be performed	Mammography—Individualization	Clinical breast examination / Mammography
**Screening intervals**	NR	Annual / Every 1–2 years	Annual	Annual	Annual	Annual	Biennial	NR	Either biennial or triennial over annual	Annual or biennial	NR	NR
	**50–69 years**											
**Screening methods**	Mammography ▪ Strong recommendation in well-resourced settings ▪ Conditional recommendation in limited resource settings	Clinical breast examination Mammography	Mammography	Mammography	Mammography (DBT preferred modality)	Clinical breast examination / Mammography (DBT is recommended, if available)	Mammography	Mammography	Mammography	Mammography	Mammography	Clinical breast examination until age of 64Mammography
**Screening intervals**	Biennial	Annually / Every 1–2 years, every 2 years after age of 55 years	Annual	≥55 years should transition to biennial screening or could continue screening annually	Annual	Annual	Biennial	Biennial or triennial	Biennial over triennial against annual	Annual or biennial	Biennial	NR
	**70–74 years**											
**Screening methods**	(70–75 years) Mammography ▪ Well-resourced settings: only for rigorous research ▪ Limited resource settings: not recommended	Clinical breast examination / Mammography	Mammography	Mammography	Mammography (DBT preferred modality)	Clinical breast examination / Mammography (DBT is recommended, if available)	Mammography	Mammography	Mammography	Mammography	Mammography	Mammography
**Screening intervals**	NR	Annual / Every 2 years after age of 55 years	Annual	≥55 years should transition to biennial screening or annually	Annual	Annual	Biennial	Biennial or triennial	Triennial over biennial	Annual or biennial	Biennial	NR
**Age to stop screening**	NR	Mammography until 75 years >75 years: decision making process	Individualization	Mammography in good health status and life expectancy of >10 years	Screening mammography should cease when life expectancy is <10 years	Consider life expectancy	Insufficient evidence to assess the balance and harms of screening mammography in women aged ≥75 years	NR	NR	NR	Insufficient evidence to assess the balance of benefits and harms of screening mammogram in women aged >75 years	NR
**Other screening methods**	40–60 years Clinical breast examination in limited resource settings	Breast self-examination is not recommended	Insufficient data to support the use of breast MRI and MBI for screening	Clinical breast examination is not recommended	Only in high-risk groups	Clinical breast examination in 25–39 every 1–3 years, >40 annually MRI is recommended in high -risk women Thermography and ductal lavage are not recommended	Insufficient evidence to assess the DBT as a primary screening method. Insufficient evidence to assess the use of U/S, MRI and DBT as adjunctive screening methods.	Use of MRI, DBT or U/S is not recommended U/S only complementary to mammography Clinical breast examination is not recommended Breast self-examination is not recommended	Screening with digital mammography alone is suggested over screening with DBT alone or with DBT in addition to digital mammography For asymptomatic women with high mammographic breast density and negative mammography results, screening with ABUS or HHUS or MRI over mammography alone is not recommended	No consensus regarding the use of U/S as a supplementary screening method	Insufficient evidence to recommend that clinical breast examination offers any benefits to women, of any age	Clinical breast examination and ultrasonography are not recommended for population-based screening

Abbreviations: ABUS: automated breast ultrasonography, ACOG: American College of Obstetricians and Gynecologists, ACR: American College of Radiology, ACS: American Cancer Society, ASBrS: American Society of Breast Surgeons, CTFPHC: Canadian Task Force on Preventive Health Care, DBT: digital breast tomosynthesis, ECIBC: European Commission Initiative on Breast Cancer, ESMO: European Society for Medical Oncology, RACGP, Royal Australian College of General Practitioners, HHUS: hand-held ultrasound, JJCO: Japanese Journal of Clinical Oncology, MBI: molecular breast imaging, MRI: magnetic resonance imaging, NCCN: National Comprehensive Cancer Network, NR: not reported, U/S: ultrasonography, USPSTF: U.S. Preventive Services Task Force, WHO: World Health Organization.

**Table 2:** Breast cancer screening recommendations in high-risk women.

	ACR	ASBrS	NCCN	ESMO
**Country**	United States of America	United States of America	United States of America	Europe
**Issued**	2018	2019	2022	2019
**Title**	Breast Cancer Screening in Women at Higher-Than-Average Risk: Recommendations From the ACR	Position Statement on Screening Mammography	Breast Cancer Screening and Diagnosis	Early breast cancer: ESMO Clinical Practice Guidelines for diagnosis, treatment, and follow-up
**Pages**	7	10	89	12
**References**	NR	24	222	107
	**A lifetime risk of breast cancer >20% based on models largely dependent on family history**			
**Recommendations for screening starting age, methods and intervals**	NR	▪ Annual mammography and access to supplemental imaging (MRI preferred modality), starting at age 35 when recommended by the physician	▪ Clinical encounter, every 6–12 months, beginning when identified as being at increased risk, but not prior to age 21 ▪ Annual mammography (tomosynthesis is recommended, if available), beginning 10 years prior to when the youngest family member was diagnosed with breast cancer, not prior to age 30, or begin at age 40 (whichever comes first) ▪ Annual MRI, beginning 10 years prior to when the youngest family member was diagnosed with breast cancer, not prior to age 25, or begin at age 40 (whichever comes first)	NR
	**History of thoracic irradiation between the ages 10 to 30 years**			
**Recommendations for screening starting age, methods and intervals**	▪ Annual DM, with or without DBT, beginning at age 25 or 8 years after radiation therapy, whichever is later	▪ Annual MRI, starting at age 25 ▪Annual mammography starting at age 30	▪ Current age < 25 years → annual clinical encounter, beginning 8 years after RT ▪ Current age ≥ 25 years→ ➢ Clinical encounter every 6–12 months, beginning 8 years after RT ➢ Annual mammography (tomosynthesis is recommended, if available), beginning 8 years after RT, but not prior to age 30 ➢ Annual MRI, beginning 8 years after RT, but not prior to age 25	NR
	**A lifetime risk of breast cancer >20% based on personal history of LCIS or ADH/ALH**			
**Recommendations for screening starting age, methods and intervals**	▪ Consider MRI	NR	▪ Clinical encounter, every 6–12 months, beginning at diagnosis of ADH or LCIS/ALH ▪ Annual mammography (tomosynthesis is recommended, if available), beginning at diagnosis of ADH or LCIS/ALH, but not prior to age 30 ▪ Consider annual MRI, beginning at diagnosis of ADH or LCIS/ALH, but not prior to 25 years	NR
	**Known genetic predisposition of breast cancer**			
**Recommendations for screening starting age, methods and intervals**	▪ Annual MRI, starting at 25 years ▪ Annual DM, with or without DBT, beginning at 30 years	▪ Annual MRI, starting at age 25 ▪ Annual mammography starting at age 30	NR	▪ Annual mammography + MRI

Abbreviations: ACR: American College of Radiology, ADH: atypical ductal hyperplasia, ALH: atypical lobular hyperplasia, ASBrS: American Society of Breast Surgeons, BRCA: breast cancer gene, DBT: digital breast tomosynthesis, DM: digital mammography, ESMO: European Society for Medical Oncology, LCIS: lobular carcinoma in situ, MRI: magnetic resonance imaging, NCCN: National Comprehensive Cancer Network, NR: not reported, PALB2: partner and localizer of breast cancer 2, RT: radiotherapy.

## Data Availability

All the data used for this article are publicly available.
